# Linking Striatal Dopaminergic Asymmetry with Personality Traits: Insights from Gambling Disorder

**DOI:** 10.1007/s10899-024-10311-9

**Published:** 2024-05-16

**Authors:** Francesco Di Carlo, Mauro Pettorruso, Mario Santorelli, Fabrizio Cocciolillo, Giacomo d’Andrea, Marco Di Nicola, Stefano S. Sensi, Giovanni Martinotti, Jon E. Grant, Giovanni Camardese, Daniela Di Giuda

**Affiliations:** 1 Department of Neuroscience, Imaging and Clinical Sciences, “G. d’Annunzio” University of Chieti, Chieti, Italy; 2 School of Medicine and Surgery, University of Milano Bicocca, Milan, Italy; 3 Institute of Nuclear Medicine, Fondazione Policlinico Universitario “A. Gemelli” Università Cattolica del Sacro Cuore, Rome, Italy; 4 Institute of Psychiatry and Psychology, Fondazione Policlinico Universitario “A. Gemelli” Università Cattolica del Sacro Cuore, Rome, Italy; 5 Center for Advanced Studies and Technology - CAST, University “G. d’Annunzio” of Chieti, Chieti, Italy; 6 Psychopharmacology, Drug Misuse and Novel Psychoactive Substances Research Unit, School of Life and Medical Sciences, University of Hertfordshire, Hatfield, UK; 7 Department of Psychiatry & Behavioral Neuroscience, University of Chicago, Chicago, IL USA

**Keywords:** Gambling disorder, Behavioural addiction, Temperament, Dopamine transporter, Basal ganglia, Neuroimaging

## Abstract

The role of dopamine in the pathophysiology of gambling disorder (GD) remains incompletely understood, with disparate research findings concerning presynaptic and postsynaptic structures and dopaminergic synthesis. The aim of this study was to investigate potential correlations between striatal dopamine transporter (DAT) lateralization and asymmetry index, as assessed by ^123^I-FP-CIT SPECT, and temperamental traits, as measured by Cloninger’s Temperament and Character Inventory (TCI), in GD subjects. Significant associations were found between DAT binding asymmetries in the caudate and putamen and the temperamental dimensions of harm avoidance and novelty seeking. Specifically, high novelty seeking scores correlated with increased DAT binding in the left caudate relative to the right, whereas higher harm avoidance scores corresponded to increased DAT binding in the right putamen relative to the left. These observations potentially imply that the asymmetry in DAT expression in the basal ganglia could be an outcome of hemispheric asymmetry in emotional processing and behavioural guidance. In summary, our study provides evidence supporting the relationship between DAT asymmetries, temperamental dimensions and GD. Future investigations could be directed towards examining postsynaptic receptors to gain a more comprehensive understanding of dopamine's influence within the basal ganglia circuit in disordered gambling. If confirmed in larger cohorts, these findings could have substantial implications for the tailoring of individualized neuromodulation therapies in the treatment of behavioural addictions.

## Introduction

Personality, acting as a significant differentiating factor among individuals, is intimately associated with the underlying neurochemical substrates (Clauss et al., 2015). Cloninger's theory of personality suggests that temperament, a heritable aspect of personality, is measurable using psychometric scales such as the Temperament and Character Inventory (TCI) (Cloninger, 1986). It highlights four fundamental temperamental domains: harm avoidance, reward dependence, novelty seeking, and persistence. Research has linked these dimensions to the modulation of specific neurotransmitters, particularly dopamine, whose variations in synthesis capacity are associated with behavioural disinhibition (Cloninger et al., 1993; Wingo et al., 2016).

Gambling disorder (GD) is a prevalent behavioural addiction globally, underpinned by increasing evidence pointing to neurobiological dysfunction in regions such as the prefrontal cortex and basal ganglia (Raimo et al., 2021; Verdejo-García et al., 2008). This disorder, similar to complex conditions like alcohol dependence, exhibits a broad range of manifestations across various patient demographics, encompassing extremes of both high and low impulsivity and novelty seeking (Nordin & Nylander, 2007; Pettorruso et al., 2023). Based on this concept, it is feasible to associate early and late-onset GD with Cloninger's Type II (characterized by high novelty seeking) and Type I (defined by high harm avoidance) temperamental profiles, respectively (Janiri et al., 2007; Shin et al., 2009). The acknowledgment of such heterogeneity is crucial for further understanding of the neurobiological bases underlying addiction.

Being vital to motivation and pleasure, dopamine influences distinct personality and behavioural facets that forge individual temperament. The interaction between dopamine and GD is yet to be fully elucidated, with existing research revealing conflicting results about presynaptic, postsynaptic structures, and dopamine synthesis (Clark et al., 2018; Pettorruso et al., 2020c). These discrepancies may stem from heterogeneous imaging methodologies (i.e., functional MRI, PET, and SPECT, the latter two employing various radioligands) and studied populations (Potenza, 2018). The dopamine transporter (DAT), an integral component of the dopaminergic system, has sparked considerable interest due to its implications in GD. Recent findings have demonstrated a relationship between reduced DAT availability and GD (Pettorruso et al., 2019b), thus suggesting that changes in DAT distribution and lateralization might affect reward preference and risk propensity (Guerra et al., 2023). While significant DAT asymmetries have been related to Parkinson's Disease (PD) (Cilia et al., 2010; Di Giuda et al., 2012), the role of physiological lateralization of DAT in non-Parkinsonian individuals remains a widely unexplored field (Garrido et al., 2020).

The existing literature on the subject is limited (Guerra et al., 2023; Kaasinen et al., 2023; Pettorruso et al., 2019b), and does not allow for a clear identification of the dopaminergic pathways underlying the different temperamental aspects of GD. Therefore, this study aimed at investigating potential correlations between striatal DAT asymmetry index and lateralization, as evaluated by ^123^I-FP-CIT SPECT imaging, and temperamental dimensions in GD patients.

## Materials and Methods

### Participants

Ten treatment-seeking male patients suffering from GD were selected from a previous study (Pettorruso et al., 2019b). They were recruited at the Addiction Unit, Fondazione Policlinico Universitario “A. Gemelli” IRCCS, Catholic University of the Sacred Heart, Rome, Italy. Inclusion criteria were the following: (i) age between 18 and 65; (ii) diagnosis of GD according to the Diagnostic and Statistical Manual of Mental Disorders, Fifth Edition (DSM‐5); (iii) active gambling, considered as a maximum of two months elapsing since last gambling session. Exclusion criteria were based on: (i) any medication interfering with DAT binding; (ii) mental impairment or documented IQ ≤ 70; (iii) significant or unstable medical and/or neurological conditions; (iv) comorbid psychiatric disorders (i.e., schizophrenia, bipolar disorder, and major depressive disorder); (v) current or past alcohol and/or substance abuse.

### Procedures

Among the self-referred treatment-seeking patients, those who met the eligibility criteria were invited to participate in the study. The project was explained in detail, and written informed consent was obtained. After the collection of general clinical and sociodemographic data (visit 1), all patients were administered psychometric testing and underwent ^123^I-FP-CIT SPECT imaging (visit 2).

The following psychometric tests were employed:

- the Yale‐Brown Obsessive–Compulsive Scale modified for GD (PG-YBOCS). The PG-YBOCS assesses the severity of gambling symptoms (behaviour and urges) during the past 7 days. Specifically, the total score can be interpreted as follows: 0–7 sub-clinical, 8–15 mild, 16–23 moderate, 24–31 severe, and 32–40 extreme gambling symptoms (Pallanti et al., 2005);
- the Gambling Severity Assessment Scale (G‐SAS). It is a 12-item self-rated scale designed to assess the severity of gambling symptoms and change during treatment. Each item has a score ranging from zero to four, and a total score is then calculated. All items request information on the past 7 days (Kim et al., 2009);
- the Timeline Followback (TLFB) interview for gambling. It is a structured interview to assess quantitative information about gambling behaviours (Weinstock et al., 2004);
- the Cloninger’s Temperament and Character Inventory (TCI). The TCI assesses four temperament dimensions: harm avoidance (HA), i.e., behavioural inhibition; novelty seeking (NS), i.e., behavioural activation; reward dependence (RD), i.e., behavioural adjustment; persistence (P), i.e., behavioural maintenance. It also evaluates three character dimensions: self-directedness (SD); cooperativeness (C); self-transcendence (ST) (Fukuhara-Makiyama et al., 2021). In particular, temperament traits are thought/believed to be influenced by genetic factors, whereas character traits by environmental factors (Pettorruso et al., 2020b). The possible answers for all the 240 items of the TCI are "yes" or "no."

### SPECT Imaging

SPECT imaging was performed as published previously (Pettorruso et al., 2019b). 185 MBq of ^123^I-FP-CIT (DaTSCAN™, G.E. Healthcare, United Kingdom) were intravenously administered 30 minutes after thyroid blockade. Acquisition was carried out 180 minutes after the radiotracer injection using a dual-head gamma camera system (E.CAM; Siemens Medical Systems, Germany) equipped with high-resolution, low-energy, parallel hole collimators. Data reconstruction was performed using filtered back projection (Butterworth filter, cut-off frequency: 0.45 cycle/cm, order 8) and Chang’s first-order attenuation correction (correction coefficient: 0.11 cm^−1^) was applied. Reconstructed slices were reoriented according to the plane connecting the frontal and occipital poles. Then, a semi-quantitative assessment was carried out using Statistical Parametric Mapping 8 (SPM8) (Wellcome Department of Cognitive Neurology, University College London, United Kingdom) for the spatial normalization of SPECT data, and the MarsBaR toolbox (version 0.43) (Ward et al., 2005) for a volume of interest (VOI) analysis within SPM. As specified earlier (Pettorruso et al., 2019b), an own DAT template was generated with the normal parametric images of 17 healthy subjects according to the method suggested by Kas et al. (2007). The ^123^I-FP-CIT images were registered with the template using the normalization algorithm provided by SPM8. A correction for the partial volume effect (PVE) was obtained by importing all normalized images in PMOD version 3.6 (PMOD Technologies Ltd, Zürich, Switzerland). Specific to non-specific ^123^I-FP-CIT binding ratio (SBR) was calculated through VOIs placed over the bilateral caudate and putamen (as radiotracer specific binding), and over the occipital cortex (as non-specific binding) of the normalized images. The VOIs were selected from a digital atlas resulting from an automatic anatomical segmentation of the spatially normalized, single subject, high‐resolution T1 MRI data set provided by the Montreal Neurological Institute (MNI) (Tzourio-Mazoyer et al., 2002). The mean counts per pixel were extracted from each VOI for all normalized PVE-corrected images using the MarSbaR toolbox. Thus, SBRs in the bilateral caudate and putamen were calculated as follows: [(mean counts in striatal VOI) − (mean counts in occipital VOI)]/(mean counts in occipital VOI).

Accordingly to studies on PD patients, DAT lateralization was calculated with the formula: *SBR left – SBR right* (Roussakis et al., 2021)*.* DAT asymmetry index was calculated with the formula: *[(SBR left—SBR right) / (SBR left* + *SBR right)]* × *100* (Fiorenzato et al., 2021; Kaasinen, 2016). Negative results indicated right asymmetry, whereas positive results left asymmetry.

### Statistical Analysis

Statistical analysis was performed using SPSS for Windows, Versions 25.0 (SPSS Inc, Chicago, Illinois). All continuous and categorical variables were expressed as mean ± standard deviation (SD) and percentage of the total, respectively, except for asymmetry index and laterality which were represented as median and range. Spearman's rank correlation coefficient was used to explore possible associations between laterality, asymmetry index and TCI subscales. All tests were two‐tailed, with statistical significance set at *p* < 0.05.

### Ethics

The study was performed in accordance with the Declaration of Helsinki and was approved by the Ethics Committee of the Catholic University of the Sacred Heart, Rome, Italy (Protocol Number: 16113/13). All patients gave their written informed consent, after a complete description of the study was provided.

## Results

Sociodemographic and clinical data of GD patients are shown in Table 1. At TLFB interview participants reported 8.00 ± 9.92 days of gambling in the previous month (median = 3.5) and 15.10 ± 12.39 days elapsing from the last gambling session. Mean scores on the G‐SAS and PG‐YBOCS scales were 25.0 ± 13.41 and 21.8 ± 7.71, respectively. TCI scores are detailed in Table 1.

**Table 1:** Sociodemographic characteristics and clinical data of the sample (n = 10)

Sociodemographic	
Age, years	49.8 (12.3)
Gender, M	10 (100%)
Education, years	9.2 (2.2)
Gambling severity	
Abstinence from gambling, days	15.1 (12.4)
	(0 – 40)
Gambling in the last month, days	8.0 (9.9)
	3.5 (0 – 30)
G-SAS	25.0 (13.4)
PG-YBOCS	21.8 (7.7)
TCI scores	
Novelty seeking (NS)	23.8 (3.8)
Harm avoidance (HA)	16.4 (4.6)
Reward dependence (RD)	13.7 (4.1)
Persistence (P)	4.7 (1.2)
Self-directedness (SD)	20.7 (3.8)
Cooperativeness (C)	27.4 (5.2)
Self-transcendence (ST)	17.9 (7.3)

Data are reported as mean (SD), median (range) and n (%), as appropriate

**Fig. 1:**
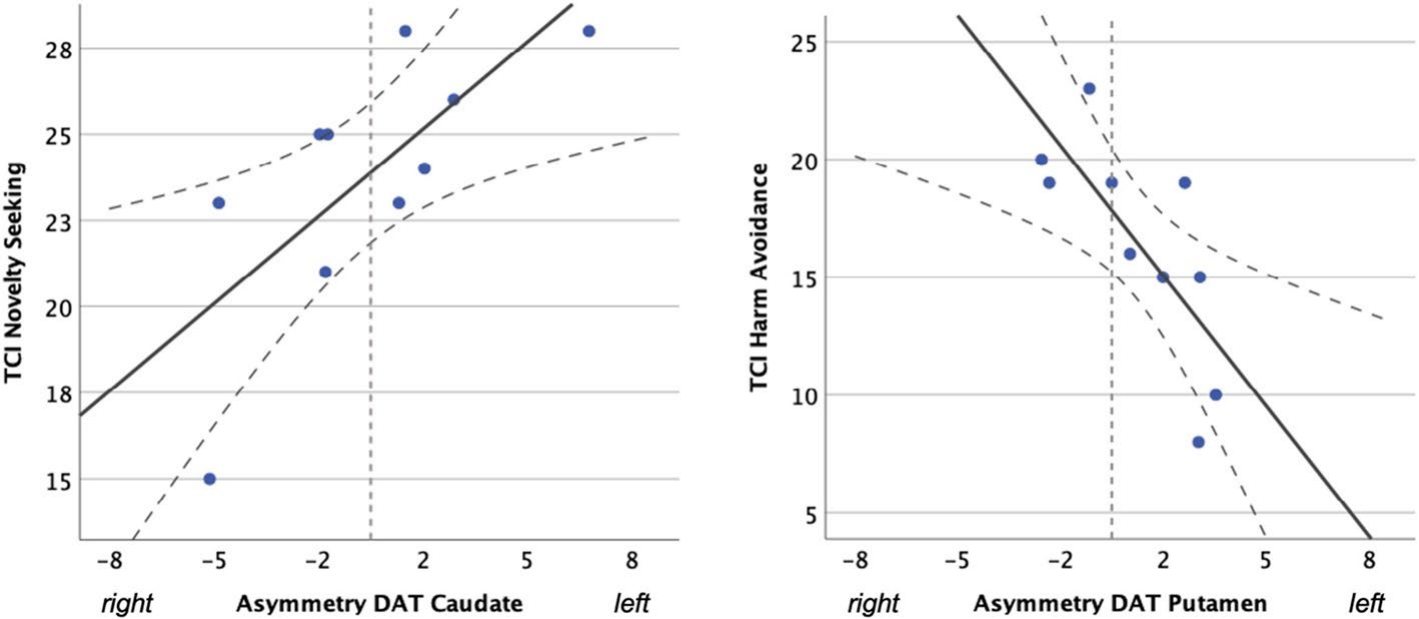
Spearman’s correlation coefficients quantifying the relationship between DAT asymmetry index in striatal regions (caudate and putamen) and Temperament and Character Inventory (TCI) traits (novelty seeking and harm avoidance)

Laterality and asymmetry index are reported in Table 2, while their correlations with TCI temperamental dimensions are detailed in Table 3. Positive correlations were found between NS scores and laterality and asymmetry index in the caudate (r = 0.709, *p* = 0.022; r = 0.734, *p* = 0.016, respectively). Conversely, negative correlations were found between HA scores and laterality and asymmetry index in the putamen (r = -0.856, *p* = 0.002; r = -0.825, *p* = 0.003, respectively)  (see also Fig. 1).

**Table 2:** DAT binding in the caudate and putamen, with laterality and asymmetry index

Specific to non-specific binding ratio (SBR)	Mean (SD)
Right caudate	377.6 (59.2)
Left caudate	377.7 (63.0)
Right putamen	305.3 (53.7)
Left putamen	310.2 (56.0)
Asymmetry index (%)	Median (range)
Caudate	-0.214 (-4.63; + 6.27)
Putamen	1.015 (-2.05; + 3.04)
Laterality	
Caudate	-2.50 (-33; + 44)
Putamen	7.00 (-11; + 22)

Volumes of interest expressed as mean (SD); laterality and asymmetry index expressed as median (range)

**Table 3:** Correlations between DAT laterality and asymmetry index, and TCI temperamental dimensions

	Asymmetry index caudate	Laterality caudate	Asymmetry index putamen	Laterality putamen
NS-TCI	0.734* (0.016)	0.709* (0.022)	-0.532 (0.113)	-0.438 (0.206)
HA-TCI	0.542 (0.106)	0.534 (0.112)	-0.825** (0.003)	-0.856** (0.002)
RD-TCI	0.226 (0.530)	0.212 (0.557)	-0.024 (0.947)	0.019 (0.959)
P-TCI	-0.169 (0.641)	-0.204 (0.572)	-0.256 (0.475)	-0.108 (0.767)

*Statistics* Spearman’s correlation coefficient, *NS* novelty seeking, *HA* harm avoidance, *RD* reward dependence, *P* persistence

## Discussion

The present study investigated the potential relationships between striatal DAT lateralization, asymmetry index, assessed by ^123^I-FP-CIT SPECT, and temperamental dimensions measured by TCI in subjects diagnosed with GD. The results revealed interesting correlations between DAT binding asymmetry in the caudate and putamen and the temperament dimensions of harm avoidance and novelty seeking. More specifically, higher novelty seeking scores coincided with increased DAT binding in the left caudate compared to the right. Conversely, higher harm avoidance scores corresponded with increased DAT binding in the right putamen relative to the left.

Over the years, numerous attempts have been made to decipher the intricate relationship between neuronal pathways and personality traits. Cloninger initially proposed three personality dimensions — harm avoidance, novelty seeking, and reward dependence — which he posited were influenced by imbalances in the serotonin, dopamine, and noradrenaline systems, respectively (Cloninger, 1986). As far as the authors are aware, this study represents the first exploration of the potential interaction between temperamental dimensions and DAT availability in the basal ganglia of GD patients.

Dopamine transporters, acting as the gatekeepers of the dopaminergic synaptic function, regulate the reuptake of the extracellular neurotransmitter by presynaptic neurons (Vaughan & Foster, 2013). The caudate nucleus, an integral part of numerous neural pathways, interacts extensively with the dorsolateral prefrontal cortex (DLPFC), which modulates cognitive and emotional control (Koechlin et al., 2003; Rahm et al., 2013). Increased DAT expression could suggest heightened dopamine availability and greater activation of the DLPFC-basal ganglia pathway.

Harm avoidance, an inheritable predisposition towards behaviour inhibition in response to signals of punishment or non-reward, is distinguished by fear of uncertainty, social inhibition, shame, and pessimistic worry in anticipation of problems (Petrosini et al., 2017). Our findings suggest that enhanced function of the right caudate might correspond to hyperactivation of the DLPFC-putamen-basal ganglia-thalamus pathway, potentially resulting in over-activation of the right hemisphere. This over-activation may serve to mitigate emotional “noise”, fear of action, and avoidance of problem-solving in the right hemisphere.

Novelty seeking, in contrast, reflects a predisposition towards exploratory activity in response to novelty, impulsive decision-making, and quick loss of temper (Petrosini et al., 2017). Analogously, the activation of the left caudate and subsequent left basal ganglia hyperactivation could be associated with the hyperactivation of the DLPFC-caudate-basal ganglia-thalamus circuits. This might result in the initiation of left cerebral pathways primarily involved in impulsive and fearless behaviours. Persistent activation in the left hemisphere could amplify the ipsilateral basal ganglia circuit, reducing the background noise of impulsivity.

Therefore, we suggest that the observed DAT asymmetry in the basal ganglia is a direct manifestation of the hemispheric asymmetry in emotion perception and behavioural guidance. Long-term stimulation of the basal ganglia on one side of the brain leads to a surge in dopamine levels in the caudate, which in turn, triggers an upregulation of dopamine transporters, key players in regulating neurotransmitter quantities in the synaptic cleft.

In line with our theoretical proposal, numerous studies have highlighted the substantial role of basal ganglia asymmetry in temperament dimensions, specifically in harm avoidance and novelty seeking (Laricchiuta et al., 2014). Indeed, in studies focused on DAT levels in PD, researchers observed a decrease in novelty-seeking behaviours among PD patients compared to healthy controls. This was particularly pronounced in instances where there was a more significant reduction of dopamine in the left hemisphere. On the other hand, higher levels of harm avoidance were found only among PD patients exhibiting reduced dopamine levels in the right hemisphere when compared to healthy controls (Porat et al., 2014; Tomer & Aharon-Peretz, 2004).

In a study by Liang et al. (2017) that involved opioid-dependent patients, an inverted-U-shaped correlation was identified between the personality trait of novelty seeking and DAT availability. Concurrently, a clinical study conducted by Menza et al. (1995) involving nine PD patients reported a positive association between novelty seeking scores and [18F]dopa uptake in the left caudate, an association that was not observed in the right caudate.

Collectively, these findings lend credence to the theoretical proposition that each hemisphere specializes in distinct aspects of emotion and behaviour regulation. The left hemisphere seems to primarily oversee motivation control and cognitive approaches pertaining to behaviour. Increased activity in the left frontal region has been linked to extroversion and sociability (Pujol et al., 2000). In contrast, a larger right anterior cingulate area has been associated with the harm avoidance dimension of temperament (Pujol et al., 2002).

Interestingly, these findings introduce the compelling prospect of dopamine-related personality traits significantly impacting the outcomes of neuromodulation treatments (Pettorruso et al., 2020a). Such treatments are typically applied to unilateral target regions (Pettorruso et al., 2021) and have been proven to effectively modulate both dopamine levels (Strafella et al., 2001) and DAT (Pettorruso et al., 2019a; Xu et al., 2023) in striatal regions. The observed asymmetries in striatal dopaminergic transmission, which run parallel to the range of temperamental facets in GD, provide a promising basis for potential advancements in personalized therapies (Pettorruso et al., 2024). Consequently, these therapeutic interventions could be diversified and tailored to individual patients, based on their distinct neurobiological profiles (Spano et al., 2019). Future research are positioned to probe deeper into this hypothesis, thereby shedding light on the heterogeneous responses to neuromodulation exhibited by patients with GD, and addictions in general (Martinotti et al., 2019, 2022).

Despite these encouraging findings, the current study does present with several limitations. A critical issue to consider is the limited sample size, which diminishes the robustness and statistical power of the findings (de Winter et al., 2016; Schönbrodt & Perugini, 2013). Moreover, the absence of a control group also constrains our ability to predict the impact of addiction on the observed findings. Additionally, the assessment of the dopaminergic synapses in the basal ganglia was exclusively carried out through DAT, a presynaptic transporter involved in the reuptake of neurotransmitters in the synaptic cleft. In the future, expanding the scope of this initial study to encompass the analysis of postsynaptic receptors could enhance our understanding of the role dopamine plays in the basal ganglia circuit.

## Conclusions

In conclusion, these initial results illuminate compelling associations between DAT binding asymmetries in the caudate and putamen regions and the temperament dimensions of harm avoidance and novelty seeking among individuals with GD. These correlations align with previously theorized models of hemispheric specialization pertaining to the regulation of emotions and behaviours. By delving deeper into these findings, we enhance our understanding of the intricate interplay between neurotransmitter systems, temperament traits, and neurobiological disorders like GD. Future studies are poised to examine the potential implications of these findings, particularly regarding the enhancement of treatment programs geared towards broadening the scope of precision medicine methodologies.

## Data Availability

The data that support the findings of this study are available on request from the corresponding author, Mauro Pettorruso.

## Declarations

G.M. has been a consultant and a speaker and has received research grants from Angelini, Doc Generici, Janssen-Cilag, Lundbeck, Otsuka, Pfizer, Servier, and Recordati. J. E. G. has received research grants from Janssen, Boehringer Ingelheim, and Biohaven Pharmaceuticals. He receives yearly compensation from Springer Publishing for acting as Editor-in Chief of the Journal of Gambling Studies and has received royalties from Oxford University Press, American Psychiatric Publishing, Inc., Norton Press, and McGraw Hill. The other Authors have nothing to declare.
